# Sighing for His Mother

**Published:** 1889-06-22

**Authors:** E. J. Clark


### Sighing for His Mother.

A touching incident which occurred in a London hospital

No one to kiss him, sinking and dying,
No loving mother or sister near,
Watch the poor laddie, looking so lonely,
Craving a kiss his sadness to cheer.

Soon he'd be leaving; strength was fast failing
Death's icy touch the scene would soon end
His trials; but is there no one to kiss him?
None to perform this act as a friend?

How he appealed to the kind-hearted sister—
"Kiss me," he pleaded, "soon I shall die.'
Prudence prevented her kissing the laddie;
Still, she felt grieved, and left with a sigh.

Is there no one to kiss him? Yes, at this moment
A mother, all tenderness, came to his side;
See her bend over him, lovingly saying
"My poor dying laddie," then she supplied
The kiss he so longed for, as if to her own,
Speaking in accents, touching and clear,
Just as a mother can—just as she does;
Always so ready to comfort and cheer.

A few loving words e'en from a stranger,
With kiss he desired, did pleasure impart;
Soon he was gone, but ere he departed
Someone did kiss him, cheering his heart.

Perhaps such a case is quite an exception,
But if it occurs it sure would be right
If the good sister kissed the poor laddie,
Helping to make his ending more bright.

Hardworking sisters never grow weary;
On the great day more clearly you'll see,
Kind acts are valued when the dear Saviour
Greets you by saying, "Ye did it to Me."

E. J. Clark.

A similar story is told in the life of Sister Dora.

